# Macrophomina Crown and Root Rot of Pistachio in California

**DOI:** 10.3390/plants9020134

**Published:** 2020-01-21

**Authors:** Mohamed T. Nouri, Daniel P. Lawrence, Craig E. Kallsen, Florent P. Trouillas

**Affiliations:** 1 Kearney Agricultural Research and Extension Center, Parlier, CA 93648, USA; mnouri@ucanr.edu; 2 Department of Plant Pathology, University of California, Davis, CA 95616, USA; dlawrence@ucdavis.edu; 3 University of California Cooperative Extension Kern County, Bakersfield, CA 93307, USA; cekallsen@ucanr.edu

**Keywords:** pistachio, crown rot, root rot, *Macrophomina phaseolina*, pathogenicity

## Abstract

In this study, declining pistachio rootstocks were detected in newly planted commercial pistachio orchards in Kern County, California. Symptoms were characterized by wilted foliage combined with crown rot in the rootstock. From diseased trees, 42 isolates were obtained, and all had similar cultural and morphological characteristics of *Macrophomina phaseolina*. Analyses of nucleotide sequences of three gene fragments, the internal transcribed spacer region (ITS1–5.8S–ITS2), partial sequences of β-tubulin, and translation elongation factor 1-α (*TEF1*) confirmed this identification, and 20 representative isolates are presented in the phylogenetic study. Testing of Koch’s postulates showed that *M. phaseolina*, when inoculated to stems and roots of the pistachio rootstocks using mycelial plugs or a microsclerotial suspension, is indeed pathogenic to this host. The widely used clonal University of California Berkeley I (UCBI) rootstock appeared highly susceptible to *M*. *phaseolina*, suggesting that this pathogen is an emerging threat to the production of pistachio in California. This study confirmed the association of *M. phaseolina* with the decline of pistachio trees and represents the first description of this fungus as a crown rot-causing agent of pistachio in California.

## 1. Introduction

Pistachio (*Pistacia vera* L.) is one of California’s many important, high-value nut crops. In 2017, California accounted for more than 99.1% of the United States pistachio crop, with approximately 101,327 hectares of bearing trees with a value of approximately $3.6 billion (http://www.acpistachios.org). Although pistachio cultivation in California is relatively new, this industry has had a record of steady expansion. As pistachio demand and acreage continues to grow, fungal pathogens pose a continually evolving challenge for the industry.

Panicle and shoot blight of pistachio has been one of the main fungal diseases affecting pistachio in California, with early reports dating back to the 1980s [1]. In California, at least eight species of Botryosphaeriaceae fungi have been associated with this disease. Botrytis blossom and shoot blight caused by *Botrytis cinerea* Pers. [2] and Alternaria late blight caused by multiple species of *Alternaria* Nees [3] are additional important diseases of pistachio in California. Management of these various diseases has mainly relied upon the use of synthetic fungicides. Additional above-ground disease of pistachio includes Cytospora canker caused by several *Cytospora* spp. and Botryosphaeria canker caused by *Neofusicoccum mediterraneum* Crous, M.J. Wingf. and A.J.L. Phillips [4,5].

Verticillium wilt caused by *Verticillium dahliae* Kleb. has been the most important soil-borne disease affecting pistachio trees in California [6]. *Verticillium dahliae* is found worldwide in all types of soils and has an extremely wide host range, affecting more than 400 different plant species, including vegetables, flowers, fruit crops, ornamentals, and perennial agronomic crops [7,8,9]. Verticillium wilt of pistachio mostly occurred in orchards planted after crops such as cotton and tomato, which are highly susceptible to *V. dahliae*, or in orchards established adjacent to fields with susceptible crops [10]. The fungus can survive in the soil for many years due to extremely persistent resting structures, the microsclerotia. Accordingly, control of Verticillium wilt begins before the trees are planted and the site location is an important consideration. Former cotton, tomato, and alfalfa fields should be avoided, as they increase the risk of Verticillium wilt. The most effective control measures to combat Verticillium wilt is the use of resistant rootstocks. In California, commercial pistachio nut production began in the 1970s with *Pistacia atlantica* Desf. as the main pistachio rootstock [11]. However, *P. atlantica* is highly susceptible to infection by *V. dahliae*, and the fungus is widespread in soils of the San Joaquin Valley. The development and widespread planting of resistant University of California Berkeley I (UCBI) rootstocks has diminished the Verticillium wilt problem of pistachio in California. Symptoms of Verticillium wilt are now only observed sporadically in the southern half of the San Joaquin Valley, where the *P. atlantica* rootstock was initially planted.

California pistachio is also subject to root and crown rots caused by oomycetes such as *Phytophthora* and *Phytopythium* [12,13,14]. Disease development with oomycetes is generally enhanced in poorly drained soils, where orchards receive long durations of flood irrigation, or in trees in lower areas or along creeks and natural drainage creeks in the orchard [6]. When the soil remains saturated for a long time, these pathogens are able to infect susceptible roots.

Crespo et al. (2019) recently revealed species of *Fusarium* and *Neocosmospora* from declining pistachio rootstocks and stem cankers in the southern San Joaquin Valley of California that were pathogenic to this host [15]. Three *Fusarium* (*Fusarium equiseti* (Corda) Sacc., *F. oxysporum* Schltdl., and *F. proliferatum* (Matsush.) Nirenberg), and two *Neocosmospora* species (*Neocosmospora falciformis* (Carrión) L. Lombard and Crous (syn: *Fusarium falciforme* (Carrión) Summerb. and Schroers) and *N. solani* (Mart.) L. Lombard and Crous (syn: *Fusarium solani* (Mart.) Sacc.)) were found associated with crown rot symptoms and vascular discoloration in stems of clonal UCBI rootstocks in California [15]. Necrotic root lesions and black discoloration of the root cortex, epidermis, and vascular tissues associated with cylindrocarpon-like fungi included species in *Dactylonectria*, *Neonectria*, and *Thelonectria*, which were also detected in multiple counties in California [16].

Other disease symptoms observed in recent surveys of pistachio orchards have included young pistachio trees that were collapsing, showing crown rot with black discolorations in the rootstocks. Isolation from these young declining pistachio rootstocks revealed the occurrence of a single putative pathogen tentatively identified as a member of the Botryosphaeriaceae. The aims of this study were to (1) identify and characterize the putative pathogen associated with pistachio rootstock decline based on molecular and morphological methods; (2) test the pathogenicity of the putative pathogen on the commonly planted UCBI pistachio rootstock in California.

## 2. Results

### 2.1. Field Surveys and Collection of Fungal Isolates

**Figure 1:**
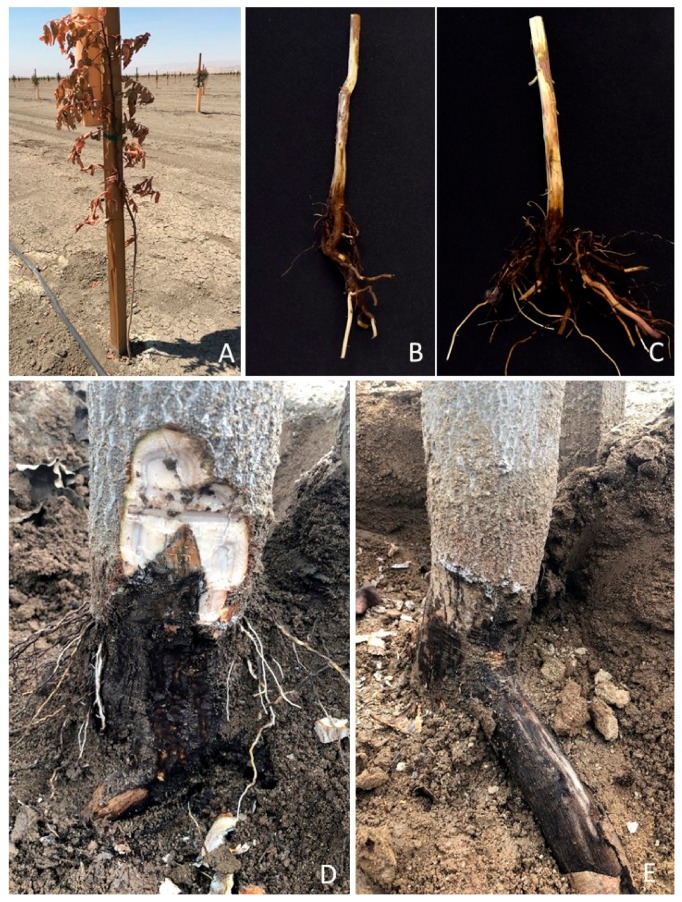
Symptoms in pistachio rootstocks associated with *Macrophomina phaseolina*; (**A**) wilting and death of unbudded University of California Berkeley I (UCBI) pistachio rootstocks; (**B**,**C**) associated crown rot symptom; (**D**,**E**) pistachio tree showing crown rot and black discoloration.

Forty-two fungal isolates resembling a member of the Botryosphaeriaceae were isolated from the rootstock of young declining pistachio trees showing root crown and lower trunk rot symptoms (Figure 1). Affected rootstocks mainly included the clonal UCBI rootstocks. All pistachio isolates were obtained from orchards in Kern County, California. Orchards characteristics included heavy clay soils and history of vegetable crops or cotton. In addition to pistachio, 21 isolates with typical characteristics of the putative pathogen were also collected: 8 from sweet cherry in Fresno and San Joaquin Counties and 13 from grapevine in Fresno County.

### 2.2. Phylogenetic Analyses

**Figure 2:**
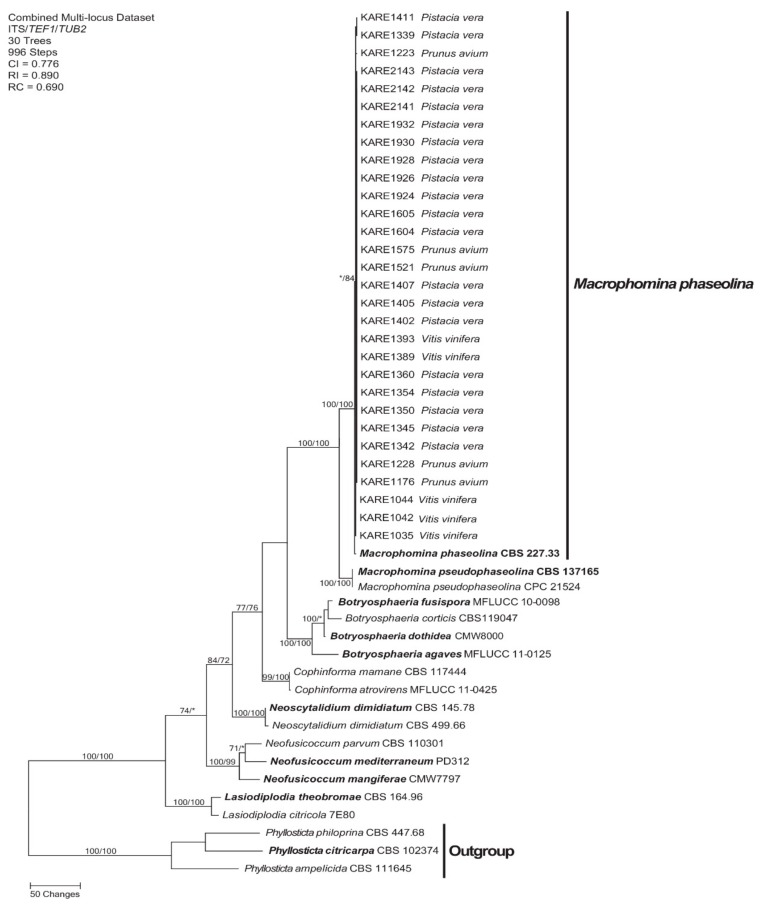
One of 30 equally most parsimonious trees resulting from the analysis of the three-gene combined dataset (ITS, *TUB2*, and *TEF1*). Numbers in front and after the slash represent maximum parsimony and maximum likelihood bootstrap values, respectively. Values represented by an asterisk were less than 70%. Scale bar represents the number of nucleotide changes.

PCR amplification of the ITS region, *TEF1*, and *TUB2* generated 452–498, 196–213, and 373–380 bp fragments, respectively. For ML analysis, the best-fit model of nucleotide evolution was K2+G for each dataset. The three-gene (ITS+*TEF1*+*TUB2*) 49 sequence dataset consisted of 1161 characters (643 characters were constant, 83 characters were parsimony-uninformative, and 435 characters were parsimony informative). MP analysis produced 30 equally most parsimonious trees of 996 steps and a consistency index (CI), retention index (RI), and rescaled consistency index (RC) of 0.776, 0.890, and 0.690, respectively. MP and ML analyses revealed that 30 Californian fungal isolates, isolated in this study, strongly clustered (100%/100% MP and ML bootstraps, respectively) with the type specimen of *Macrophomina phaseolina* (Tassi) Goid. isolate CBS 227.33 (Figure 2).

### 2.3. Morphological Characterization

**Figure 3:**
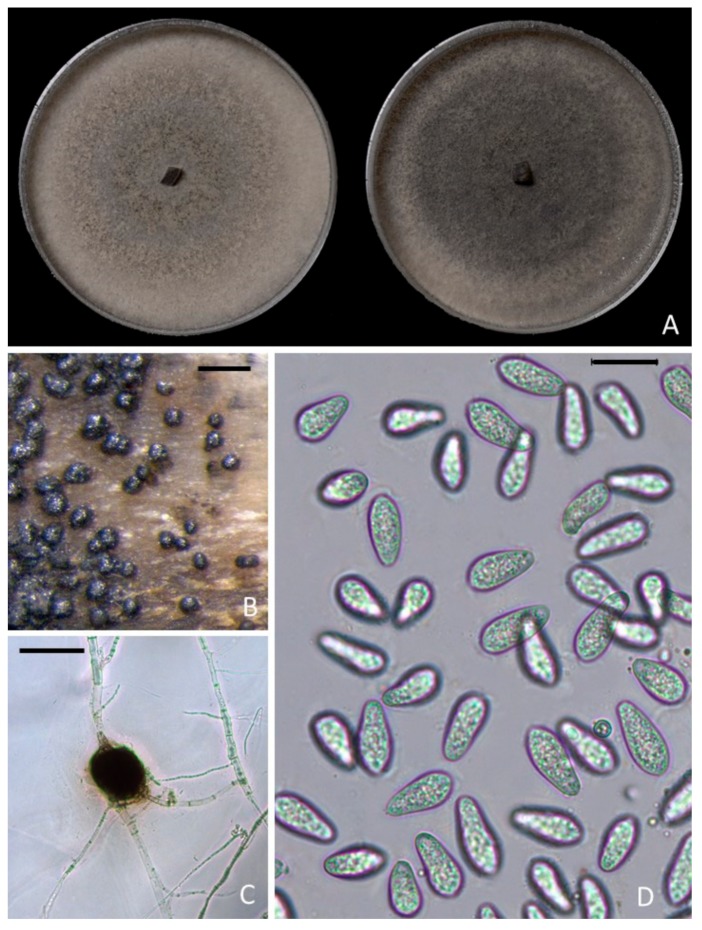
Morphological characteristics of *Macrophomina phaseolina*; (**A**) colonies on potato dextrose agar after 10 days of incubation at 25 ± 1 °C in darkness; (**B**,**C**) microsclerotia on pistachio wood and close up on a microsclerotium produced on water agar; (**D**) conidia produced from mature pycnidia forming on pistachio leaf agar medium. Scale bar in B = 300 μm; in C = 60 μm; and in D = 20 μm.

**Figure 4:**
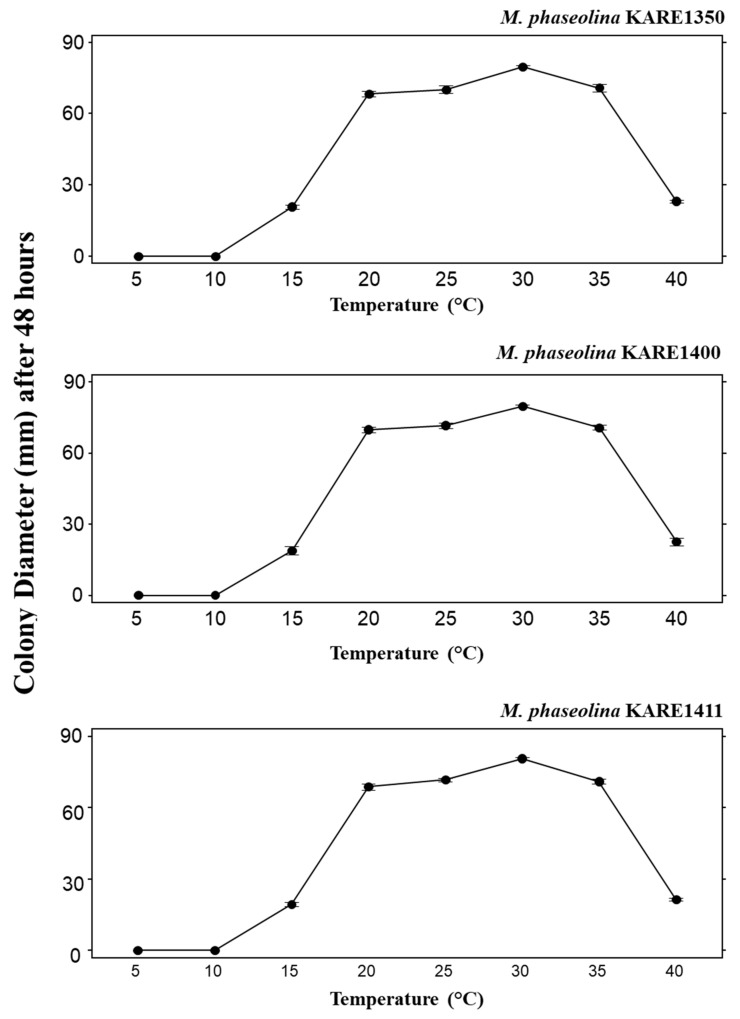
Effect of the temperature on the mycelial growth of three isolates of *Macrophomina phaseolina*: KARE1350, KARE1400 and KARE1411 on potato dextrose agar after 48 h of incubation. Error bars represent the standard error of the mean.

Colonies in culture ranged in color from light to dark gray and became black with age (Figure 3A). The average growth rate was 4 cm per day, and most colonies reached the edge of an 85 mm potato dextrose agar (PDA) dish in 48 h. Aerial mycelia generally did not develop on PDA. Hyphae were septate, initially subhyaline turning dark-brown with time. Abundant microsclerotia developed in water agar (WA) and PDA and were black, spherical to oblong, and averaged 84.5 (Length) × 53.5 (Width) μm (n = 40) with a length-width ratio of 1.57 at maturity (Figure 3B,C). Pycnidia, produced on pistachio leaf agar (PLA) medium after 2 weeks incubation, were black, subglobose to lageniform, solitary or gregarious, and ranged from 100 to 200 μm in diameter. Conidia were ellipsoid to obovoid and averaged 24.5 × 11.0 μm with a length-width ratio of 2.28. Immature conidia possessed apical mucoid appendages (Figure 3D). Morphological features of the isolates were typical of those of *M. phaseolina* [17]. The optimal growth temperature was 30 °C for all three isolates tested (Figure 4).

### 2.4. Pathogenicity Tests

#### 2.4.1. Stem Inoculation of Pistachio Rootstocks with Mycelium Plugs

**Figure 5:**
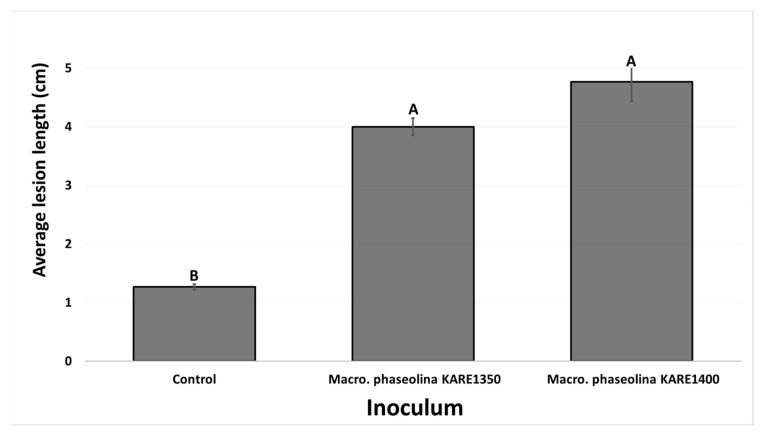
Average lesion lengths (cm) on pistachio clonal UCBI rootstocks 10 months after inoculation with mycelial plugs of *Macrophomina phaseolina* isolates KARE1350 and KARE1400 (September 2016 inoculations). Columns with different letters indicate treatment means that are significantly different (*p* = 0.0005).

Ten months after inoculation, lesion and rot symptoms observed from the inoculated stems were similar to those observed in the field. For the first pathogenicity test conducted in September 2016, the two fungal isolates tested produced dark vascular discoloration in the wood that ranged from 4 to 4.76 cm in length (Figure 5). Both isolates tested produced significantly longer (*p* = 0.0005) lesion lengths on pistachio stems compared to the control treatment (1.2 cm).

**Figure 6:**
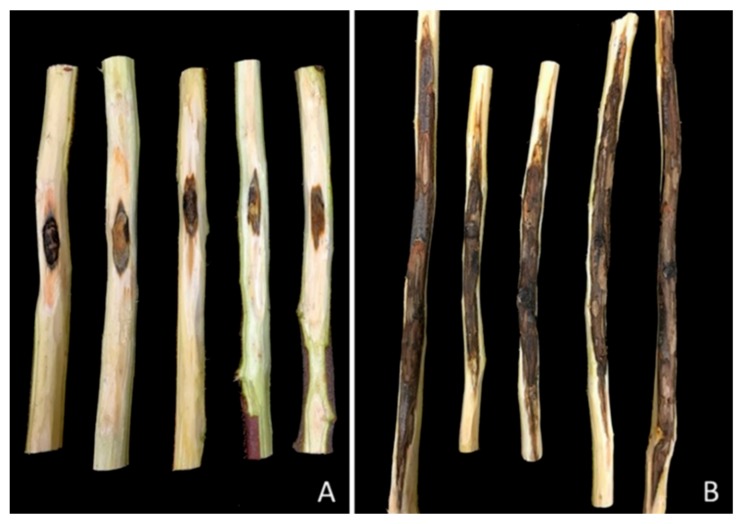
Lesions produced in 2-year-old potted clonal UCBI rootstocks, 10 months after inoculation: (**A**) Control; (**B**) *Macrophomina phaseolina* isolate KARE1400 (July 2017 inoculations).

**Figure 7:**
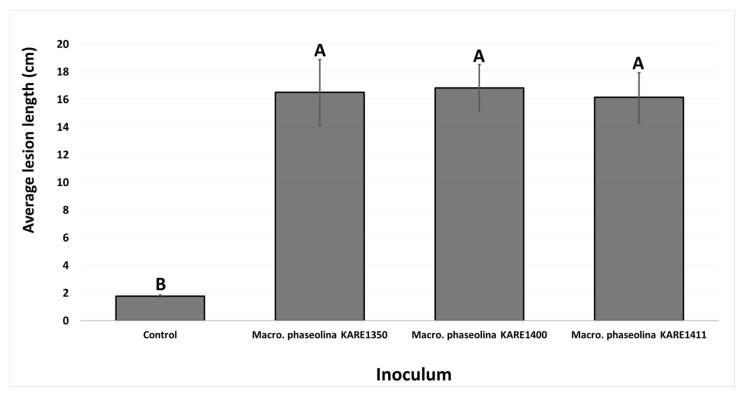
Average lesion lengths (cm) on pistachio clonal UCBI rootstocks 10 months after inoculation with mycelial plugs of *Macrophomina phaseolina* isolates KARE1350, KARE1400, and KARE1411 (July 2017 inoculations). Columns with different letters indicate treatment means that are significantly different (*p* < 0.00001).

In the second experiment, conducted in July 2017, all three isolates tested produced substantial lesions to the stems of pistachio clonal UCB1 rootstocks (Figure 6). Mean lesion length varied between 16.15 and 16.83 cm and was significantly longer (*p* < 0.00001) as compared to the control plants (2 cm) (Figure 7). Each fungal isolate was successfully recovered from inoculated plants; pathogen recovery varied between 66.6% and 100% among the three isolates, and morphologically matched the inoculated fungus, thereby fulfilling Koch’s postulates of pathogenic organisms. The controls showed no disease symptoms and no pathogen was isolated from them.

#### 2.4.2. Root Inoculation of Pistachio Rootstocks with Microsclerotial Suspension

**Figure 8:**
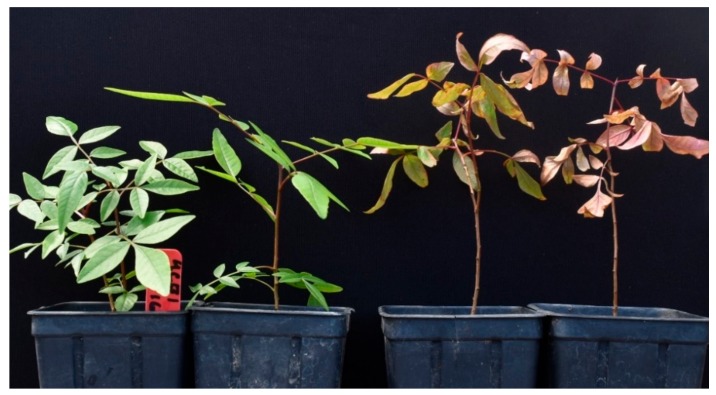
Symptoms produced on 4-month-old plantlets of clonal UCBI rootstocks after 22 days incubation and following root inoculation using a microsclerotia suspension with control plants (left) and inoculated plants (right).

Three weeks after inoculation, all isolates tested had caused severe wilting or death of all the inoculated plantlets of clonal UCBI rootstocks (Figure 8). Isolates were recovered consistently from resulting root or crown lesions thus, fulfilling Koch’s postulates. *Macrophomina phaseolina* was not isolated from control plants, which remained asymptomatic.

## 3. Discussion

This is the first study to report *M. phaseolina* associated with pistachio rootstock decline in California. *Macrophomina phaseolina* was isolated from black lesions developing at the crown or basal stem of rapidly declining young pistachio trees in Kern County California. The identification of *M. phaseolina* was supported by morphological examinations of the unique colony characteristics, including fast growth and other anamorphic morphologies typical of the species including obtuse conidia with apical mucoid appendages and the production of microsclerotia in culture [17,18].

Pathogen identification was confirmed by phylogenetic analyses of ITS, *TEF1,* and *TUB2* DNA sequence data. These analyses revealed that isolates collected from different symptomatic parts in pistachio rootstocks (crown rot and root rot) represented a single species. Results of phylogenetic analyses also indicated that *M. phaseolina* isolates from pistachio were genetically similar to other isolates isolated from symptomatic grapevine and sweet cherry in Fresno and San Joaquin Counties, respectively.

*Macrophomina phaseolina* is an important phytopathogenic fungus, infecting more than 750 plant species [17,19]. The pathogen incites a stem canker disease in many crops that is often referred to as charcoal rot disease, due to the charcoal type coloration imparted to the symptomatic plant tissues. Charcoal rot disease affects many field crops including soybean (*Glycine max* (L.) Merr.) [20], chickpea (*Cicer arietinum* L.) [21], common bean (*Phaseolus vulgaris* L.) [22], sunflower (*Helianthus annuus* L.) [23], and sorghum (*Sorghum bicolor* (L.) Moench.) [24]. In California, charcoal rot has emerged as a serious concern for strawberry cultivation [25]. By 2014, the pathogen was confirmed in all major coastal strawberry counties in California [26]. *Macrophomina phaseolina* has been reported only sporadically as a pathogen of perennial woody crops. It is known to affect grapevine in Australia, Iran, South Africa, Spain, and California [27,28,29] and olive in Australia [30]. In California, *M. phaseolina* has also been reported from almond cankers [31].

Pathogenicity tests in potted 2-year-old clonal UCBI pistachio rootstocks using mycelium plugs showed that *M. phaseolina* was highly aggressive following July infections, causing dark and elongated lesions in the stems of the pistachio trees. In the microsclerotial inoculum assay, *M. phaseolina* caused the death of clonal UCBI pistachio plantlets, inducing both root and crown rots. These results indicate that clonal UCBI pistachio rootstock most likely do not possess genetic resistance against this aggressive plant pathogen. To our knowledge, this work is the first to report *M. phaseolina* associated with the decline of pistachio trees worldwide.

*Macrophomina phaseolina* causes important annual losses to its host crop and can survive in the soil for many years, mainly as microsclerotia that germinate repeatedly during the growing season. The pathogen generally attacks young plants when their growth is retarded due to unfavorable conditions including low-water potentials that occur during periods of severe drought [32]. In the present study, *M. phaseolina* was mostly isolated from young rootstocks that were newly planted in the field and were subjected to stressful environmental conditions such as high temperature, poor soil fertility, or trees that had suffered bark damage due to gophers.

In recent years, California has been severely affected by drought and increased mean annual temperatures, which may contribute to fluxes in the plant environment and increase plant stress. Water shortage combined with high temperatures, the intensification and expansion of pistachio cultivation, the aging of orchards, and the planting of new varieties are also factors that could contribute to disease emergence and spread. Recently, multiple *Cytospora* spp., *Neofusicoccum mediterraneum*, *Colletotrichum karsti* You L. Yang, Zuo Y. Liu, K.D. Hyde and L. Cai, *Diaporthe ambigua* Nitschke, and *Didymella glomerata* (Corda) Qian Chen and L. Cai, were reported as new pathogens of pistachio in California, causing cankers in the trunks and branches of mature trees [4,5].

In conclusion, this study has provided new information regarding the identity and pathogenicity of *M. phaseolina* associated with pistachio rootstock decline in California, and this pathogen represents a new and emerging threat to the industry. The occurrence of this fungal pathogen, in pistachio but also sweet cherry and grapevine in California, has become of increasing concern in recent years. Research needs to be continued to investigate the disease epidemiology as well as develop efficient control strategies to mitigate the impact of Macrophomina crown and root rot disease of pistachio in California.

## 4. Materials and Methods

### 4.1. Field Surveys and Collection of Fungal Isolates

Isolates used in this study were isolated from declining pistachio trees and rootstocks expressing crown and root rot symptoms in Kern County California from 2016 to 2018. In total, 22 symptomatic samples were collected from 8 orchards ranging from 1- to 5-years-old. Samples of phloem (live bark) and vascular-cambium tissue from the lower trunk/crown of young trees showing crown rot and associated decline symptoms were collected and taken to the laboratory for detailed examination and isolation of putative pathogens. Symptomatic bark and root tissues were surface disinfested by submerging them in a 0.5% sodium hypochlorite for 2 min and rinsed twice with sterile water. Subsequently, the bark and root tissues were placed in Petri dishes containing potato dextrose agar (PDA) acidified with lactic acid (2.5 mL of 25% [vol/vol] per liter of medium) (APDA). Cultures were incubated at ambient laboratory light and temperature conditions (24 ± 2 °C) with approximately 12 h of daylight and 12 h of darkness until fungal colonies were observed. The most prevalent fungal taxa growing from the symptomatic tissues were then individually transferred to fresh APDA Petri dishes. To obtain pure cultures, single hyphal tips from colonies with typical growth characteristics of the Botryosphaeriaceae (initially white, fast-growing, and darkening with age) were transferred to fresh PDA and incubated as described above for 5 to 7 days. Similar isolates were also isolated from symptomatic grapevine and sweet cherry wood tissues and were included in the phylogenetic analyses. Isolates collected in the present study are summarized in Table 1 and maintained in the culture collection of the Department of Plant Pathology of the University of California, Davis at the Kearney Agricultural Research and Extension (KARE) Center, Parlier, CA, USA.

### 4.2. DNA Extraction, Sequencing, and Phylogenetic Analyses

Total genomic DNA was isolated from fungal mycelium scraped with a sterile scalpel from the surface of 7-day-old PDA cultures using the DNeasy Plant Kit (Qiagen, Valencia, CA, USA), following the manufacturer’s instructions. All PCR reactions utilized AccuPower™ PCR Premix (Bioneer, Alameda, CA, USA), following the manufacturer’s instructions. Amplification of rDNA, including the intervening ITS regions and 5.8S rDNA (ITS1–5.8S–ITS2), using the primer set ITS1 and ITS4 followed the protocol of White et al. (1990) [33]. Amplification of translation elongation factor 1-α (*TEF1*) fragments utilized the primer set EF1-728F and EF1-986R [34], and beta-tubulin (*TUB2*) utilized primers Bt2a and Bt2b [35]. PCR products were visualized on 1.5% agarose gels (120 V for 25 min) stained with GelRed^®^ (Biotium, Fremont, CA, USA), following the manufacturer’s instructions, to confirm presence and size of PCR amplicons, purified via Exonuclease I and recombinant Shrimp Alkaline Phosphatase (Affymetrix, Santa Clara, CA, USA), and sequenced in both directions utilizing the primers above on an ABI 3730 Capillary Electrophoresis Genetic Analyzer (College of Biological Sciences Sequencing Facility, University of California, Davis, CA, USA).

Forward and reverse DNA sequences were assembled, edited, and proofread in Sequencher v. 5 (Gene Codes Corporation, Ann Arbor, MI, USA) and deposited in GenBank (Table 1). Homologous sequences with high similarity from ex-type and non-type Botryosphaeriaceae species were included for phylogenetic reference utilizing the BLASTn function in NCBI and literature review (Table 2). Multiple sequence alignments were conducted in MEGA v. 6 [36] and manually adjusted where necessary in Mesquite v. 3.10 [37]. Alignments were submitted to TreeBASE under accession number S24559. The three-gene dataset was analyzed using two different optimality search criteria, maximum parsimony (MP) and maximum likelihood (ML), in PAUP * v. 4.0a164 and GARLI v. 0.951 [38,39], respectively. For the MP analysis, a heuristic search with 1000 random sequence additions was implemented with the Tree-Bisection-Reconnection algorithm and gaps were treated as missing data. Bootstrap analysis with 1000 replicates was used to estimate branch support. For the ML analysis, MEGA was used to infer a model of nucleotide substitution for each dataset, using the Akaike Information Criterion (AIC). ML analyses were conducted according to the best fit model of nucleotide substitution for each dataset using default parameters in GARLI, and branch support was determined by 1000 bootstrap replicates. Sequences of *Phyllosticta* Pers. (Botryosphaeriales, Phyllostictaceae) served as the outgroup taxon in the phylogenetic analyses.

### 4.3. Morphological Characterization

Three representative isolates (KARE1045, KARE1350, and KARE1400) were selected to study their cultural and conidial features. For each selected isolate, 5 mm mycelial plugs from developing colonies were removed and transferred to the center of new 85 mm diameter PDA Petri dishes. Cultures were incubated in the dark at temperatures ranging from 5 to 40 °C at 5-degree intervals. At each temperature (5°, 10°, 15°, 20°, 25°, 30°, 35°, and 40 °C), 3 replicate dishes of each isolate were used. Measurements of colony diameter were taken each 24 h until the fastest-growing colony had reached the edge of the dish. For each of the 8 temperatures, the average colony diameter per 48 h of growth was calculated. Colony characters and pigment production were noted after 48 h of incubation on PDA at 30 °C in darkness. The experiment was repeated once.

The representative isolates, as mentioned above, were further used to study conidial morphology. Pycnidia were induced to form by incubating colonies on pistachio leaf agar (PLA) medium [40]. Pistachio leaves were autoclaved twice at 120 °C for 20 min and then placed into Petri dishes containing PDA (5 g agar, 5 g potato dextrose broth and 500 mL water). Cultures were then incubated under near UV irradiation (12 h light/12 h dark) at 25 ± 1 °C for 2 weeks. Pycnidia containing conidia were mounted in sterile water on glass slides, and conidial dimensions included length and width of 40 conidia for each isolate were measured at ×400 magnification using a Leica compound microscope (Leica DM2000 LED Microscope, Wetzlar, Germany). Microsclerotia were also induced to form by incubating isolates on WA and characterized as described above.

### 4.4. Pathogenicity Tests

#### 4.4.1. Stem Inoculation of Clonal UCBI Pistachio Rootstocks with Mycelium Plugs

Potted 2-year-old clonal UCBI pistachio rootstocks showing no apparent decline symptoms, or weak growth, were used for the pathogenicity tests. Two experiments were conducted using the same methodology. The first pathogenicity test was conducted in September 2016. Four tree replicates were inoculated per combination of 3 inoculation treatments (isolates KARE1350, KARE1400, and the control). Plants were arranged in a completely randomized design and maintained in a greenhouse with a natural photoperiod and temperatures ranging from 22 to 30 °C for 10 months. Plants were watered once weekly during the course of the experiment. The second pathogenicity test was conducted in July 2017. Eight replicate trees were inoculated per combination of 4 inoculation treatments (isolates KARE1350, KARE1400, KARE1411, and the control) and arranged in a completely randomized design in an outdoor gravel bed for 10 months. Inoculations were conducted by using a 5 mm-diameter mycelium plug from a 7-day-old culture. The bark surface was disinfected at the point of inoculation with 70% ethanol. Stems of the potted clonal UCBI rootstocks were inoculated 4 cm above the soil line, wounds to the stem/crown were produced with a flame-sterilized 5 mm cork borer followed by placing a 5 mm agar plug bearing aerial mycelia, upper surface facing inward, into the fresh wound, which was then sealed with petroleum jelly, and wrapped with Parafilm. Control plants were treated with sterile PDA agar plugs followed by sealing and wrapping as above. Plants were watered twice weekly.

Infection data were recorded 10 months after inoculation. The bark was removed, and the length of wood discoloration (LWD) upwards and downwards from the point of inoculation was measured using a digital caliper. In an attempt to fulfill Koch’s postulates, small pieces of necrotic tissue from the edge of each lesion were surface disinfested as described above and plated on APDA filled Petri dishes to recover the inoculated fungus. Emerging fungal colonies were recorded and identified based on morphology as described above. Data for lesion length were tested for normality and homogeneity of variance using Shapiro-Wilk’s and Bartlett’s tests, respectively. ANOVAs were done in Statistix 10 (Analytical Software, Tallahassee, FL, USA) to evaluate differences in the LWD between the control and fungal treatments. Differences in the means between fungal treatments and the mock-inoculated controls were assessed using Tukey’s honestly significant difference (HSD) test at *p* = 0.05.

#### 4.4.2. Root Inoculation of UCBI Pistachio Rootstocks with Microsclerotial Suspensions

Clonal UCBI pistachio saplings were root-inoculated using a microsclerotial suspension. The experiment was conducted using 3 isolates (KARE1350, KARE1400, and KARE1411) of *M. phaseolina* inoculated to 4-month-old plantlets of clonal UCBI rootstocks. The inoculum, consisting of mycelium and microsclerotia (1 × 10^5^ propagules per mL), was prepared by scraping the surface of 12-day-old PDA cultures containing microsclerotia of the pathogen with a sterile scalpel blade and homogenizing in sterile water using a kitchen blender on high speed for 30 s. Inoculations were performed by dipping the bare root system of each rootstock into the inoculum slurry for 5 min, and subsequently mixing the microsclerotia suspension homogeneously with potting soil in 10 × 24 cm tree pots. In total, 20 clonal UCBI plantlet rootstocks were used. These included 5 replicates for each fungal isolate and the control. The 5 mock-inoculated controls were subjected to the same process as described above but were treated with a mixture of distilled sterile water and potted and incubated as above. Disease development and symptoms were assessed every 4 days for 1 month.

## Figures and Tables

**Table 1:** Fungal isolates recovered in this study.

Species	Isolate ^a^	Host	Substrate	Location	GenBank Accession Number ^b^		
					ITS	TEF1	TUB2
***Macrophomina phaseolina***	**KARE1339**	***Pistacia vera***	**Crown**	Kern Co., CA	MN097202	MN106057	MN106087
*Macrophomina phaseolina*	KARE1342	*Pistacia vera*	Crown	Kern Co., CA	MN097203	MN106058	MN106088
*Macrophomina phaseolina*	KARE1345	*Pistacia vera*	Root	Kern Co., CA	MN097204	MN106059	MN106089
*Macrophomina phaseolina*	KARE1350	*Pistacia vera*	Crown	Kern Co., CA	MN097205	MN106060	MN106090
*Macrophomina phaseolina*	KARE1354	*Pistacia vera*	Crown	Kern Co., CA	MN097206	MN106061	MN106091
*Macrophomina phaseolina*	KARE1360	*Pistacia vera*	Root	Kern Co., CA	MN097207	MN106062	MN106092
*Macrophomina phaseolina*	KARE1402	*Pistacia vera*	Crown	Kern Co., CA	MN097210	MN106065	MN106095
*Macrophomina phaseolina*	KARE1405	*Pistacia vera*	Crown	Kern Co., CA	MN097211	MN106066	MN106096
*Macrophomina phaseolina*	KARE1407	*Pistacia vera*	Crown	Kern Co., CA	MN097212	MN106067	MN106097
*Macrophomina phaseolina*	KARE1411	*Pistacia vera*	Root	Kern Co., CA	MN097213	MN106068	MN106098
*Macrophomina phaseolina*	KARE1604	*Pistacia vera*	Crown	Kern Co., CA	MN097216	MN106069	MN106101
*Macrophomina phaseolina*	KARE1605	*Pistacia vera*	Crown	Kern Co., CA	MN097217	MN106070	MN106102
*Macrophomina phaseolina*	KARE1924	*Pistacia vera*	Crown	Kern Co., CA	MN097218	MN106073	MN106103
*Macrophomina phaseolina*	KARE1926	*Pistacia vera*	Crown	Kern Co., CA	>MN097219	MN106074	MN106104
*Macrophomina phaseolina*	KARE1928	*Pistacia vera*	Crown	Kern Co., CA	MN097220	MN106075	MN106105
*Macrophomina phaseolina*	KARE1930	*Pistacia vera*	Crown	Kern Co., CA	MN097221	MN106076	MN106106
*Macrophomina phaseolina*	KARE1932	*Pistacia vera*	Crown	Kern Co., CA	MN097222	MN106077	MN106107
*Macrophomina phaseolina*	KARE2141	*Pistacia vera*	Crown	Kern Co., CA	MN097223	MN106078	MN106108
*Macrophomina phaseolina*	KARE2142	*Pistacia vera*	Crown	Kern Co., CA	MN097224	MN106079	MN106109
*Macrophomina phaseolina*	KARE2143	*Pistacia vera*	Crown	Kern Co., CA	MN097225	MN106080	MN106110
*Macrophomina phaseolina*	KARE1176	*Prunus avium*	Root	San Joaquin Co., CA	MN097199	MN106054	MN106084
*Macrophomina phaseolina*	KARE1223	*Prunus avium*	Root	San Joaquin Co., CA	MN097200	MN106055	MN106085
*Macrophomina phaseolina*	KARE1228	*Prunus avium*	Root	San Joaquin Co., CA	MN097201	MN106056	MN106086
*Macrophomina phaseolina*	KARE1521	*Prunus avium*	Root	Fresno Co., CA	MN097214	MN106071	MN106099
*Macrophomina phaseolina*	KARE1575	*Prunus avium*	Root	Fresno Co., CA	MN097215	MN106072	MN106100
*Macrophomina phaseolina*	KARE1035	*Vitis vinifera*	Trunk	Fresno Co., CA	MN097196	MN106051	MN106081
*Macrophomina phaseolina*	KARE1042	*Vitis vinifera*	Cordon	Fresno Co., CA	MN097197	MN106052	MN106082
*Macrophomina phaseolina*	KARE1044	*Vitis vinifera*	Cordon	Fresno Co., CA	MN097198	MN106053	MN106083
*Macrophomina phaseolina*	KARE1389	*Vitis vinifera*	Trunk	Fresno Co., CA	MN097208	MN106063	MN106093
*Macrophomina phaseolina*	KARE1393	*Vitis vinifera*	Trunk	Fresno Co., CA	MN097209	MN106064	MN106094

^a^ KARE = Kearney Agricultural Research and Extension. ^b^ ITS = internal transcribed spacer, *TEF1* = translation elongation factor 1α, and *TUB2* = beta-tubulin.

**Table 2:** Fungal isolates retrieved from Genbank for phylogenetic reference.

Species	Isolate ^a^	Host/Substrate	Location	GenBank Accession Number ^b^		
				ITS	*TEF1*	*TUB2*
***Botryospheria agaves***	**MFLUCC 11-0125**	***Agaves* sp.**	Thailand	JX646791	JX646856	JX646841
*Botryospheria corticis*	**CBS 119047**	*Vaccinium corymbosum*	NJ, USA	DQ299245	EU017539	EU673107
*Botryospheria dothidea*	**CMW8000**	*Prunus* sp.	Switzerland	AY236949	AY236898	AY236927
*Botryospheria fusispora*	**MFLUCC 10-0098**	*Caryota* sp.	Thailand	JX646789	JX646854	JX646839
*Cophinforma eucalypti*	MFLUCC 11-0425	*Eucalyptus* sp.	Thailand	JX646800	JX646865	JX646848
*Cophinforma mamane*	CBS 117444	*Eucalyptus* sp.	Venezuela	KF531822	KF531801	KF531802
*Lasiodiplodia theobromae*	**CBS 164.96**	Fruit on coral reef coast	New Guinea	AY640255	AY640258	EU673110
*Lasiodiplodia citricola*	7-E80	*Juglans regia*	CA, USA	KC357300	KC357312	KC357306
*Macrophomina phaseolina*	**CBS 227.33**	*Zea mays*	Palestine	KF531825	KF531804	KF531806
*Macrophomina pseudophaseolina*	**CBS 137165**	*Arachis hypogaea*	Senegal	KF951791	KF952153	KF952233
*Macrophomina pseudophaseolina*	CPC 21524	*Hibiscus sabdarifa*	Senegal	KF951799	KF952161	KF952240
*Neofusicoccum mangiferae*	CBS 118532	*Mangifera indica*	Australia	AY615186	DQ093220	AY615173
*Neofusicoccum mediterraneum*	**PD312**	*Eucalyptus* sp.	Greece	GU251176	GU251308	GU251836
*Neofusicoccum parvum*	CBS 110301	*Vitis vinifera*	Portugal	AY259098	AY573221	EU673095
*Neoscytalidium dimidiatum*	**CBS 145.78**	*Homo sapiens*	United Kingdom	KF531816	KF531795	KF531796
*Neoscytalidium dimidiatum*	CBS 499.66	*Mangifera indica*	Mali	KF531820	KF531798	KF531800
*Phyllosticta ampelicida*	CBS 111645	*Parthenocissus quinquefolia*	MO, USA	FJ824766	FJ824772	FJ824777
*Phyllosticta citricarpa*	**CBS 102374**	*Citrus aurantium*	Brazil	FJ824767	FJ538371	FJ824778
*Phyllosticta philoprina*	CBS 447.68	*Taxus baccata*	United States	FJ824768	FJ824773	FJ824779

^a^ Isolates in bold represent type specimens. ^b^ ITS = internal transcribed spacer, *TEF1* = translation elongation factor 1α, and *TUB2* = beta-tubulin.
